# Template-based breast IMRT planning for increased workload efficiency

**DOI:** 10.1186/1748-717X-8-67

**Published:** 2013-03-20

**Authors:** Sonia Kim Anh Nguyen, Fred Cao, Ramani Ramaseshan, Sarah Kristensen, Krista Kuncewicz, Vicky Huang, Craig Elith, Peter Steiner, Jennifer Hayes, Beverly Lester, Cheryl McGregor, Bilal Shahine, Winkle Kwan

**Affiliations:** 1 British Columbia Cancer Agency-Fraser Valley Centre, 13750-96th Avenue, Surrey, BC, V3V 1Z2, Canada; 2 British Columbia Cancer Agency- Abbotsford Centre, Abbotsford, BC, Canada

**Keywords:** Breast cancer, Template-based intensity-modulated radiotherapy, Radiation treatment

## Abstract

**Background:**

To be less resource intensive, we developed a template-based breast IMRT technique (TB-IMRT). This study aims to compare resources and dose distribution between TB-IMRT and conventional breast radiation (CBR).

**Methods:**

Twenty patients with early stage breast cancer were planned using CBR and TB-IMRT. Time to plan, coverage of volumes, dose to critical structures and treatment times were evaluated for CBR and TB-IMRT. Two sided-paired *t* tests were used.

**Results:**

TB**-**IMRT planning time was less than CBR (14.0 vs 39.0 min, *p* < 0.001). Fifteen patients with CBR needed 18 MV, and 11 of these were planned successfully with TB-IMRT using 6 MV. TB-IMRT provided better homogeneity index (0.096 vs 0.124, *p* < 0.001) and conformity index (0.68 vs 0.59, *p* = 0.003). Dose to critical structures were comparable between TB-IMRT and CBR, and treatment times were also similar (6.0 vs 7.8 min, p = 0.13).

**Conclusions:**

TB- IMRT provides reduction of planning time and minimizes the use of high energy beams, while providing similar treatment times and equal plans compared to CBR. This technique permits efficient use of resources with a low learning curve, and can be done with existing equipment and personnel.

## Introduction

Breast cancer is the most common invasive female cancer in North America and early stage disease comprises the majority of cases. Radiation is a crucial component of therapy for women with early stage breast cancer. With the advent of breast conservation therapy, breast cancer patients can now preserve the breast with the same survival outcomes as modified radical mastectomy. Radiation reduces breast cancer recurrence rates by two-thirds, with an associated survival benefit as well [1,2].

Radiation practice has shifted from two-dimensional therapy based on conventional simulator and anatomical landmarks to a three-dimensional approach using CT planning. Radiation advances have made way for breast intensity modulated radiation therapy (IMRT) to further improve the planning process and delivery of radiation. Early data has shown that IMRT can achieve a more homogeneous dose distribution while delivering less dose to normal tissue [3-5], which has translated into a reduction of side effects [6,7]. Over recent years, the use of breast IMRT has been increasing, at least in the United States [8] and large academic centers [6].

Nevertheless, conventional breast radiation (CBR) is still used in a larger percentage of radiation treatment facilities in North America and Europe in lieu of breast IMRT [9]. While the utility of IMRT is particularly evident when target volumes have complex shapes, or are near organs at risk, the homogeneity and toxicity advantages of IMRT for the breast have to be weighed against workload impact. Conventional tangential beams that deliver radiation to the whole breast remain simple and effective. In view of possible limited clinical resources and time constraints, newer radiation planning techniques such as IMRT may burden the health care system. This is particularly true in the setting of adjuvant breast radiation, which represents a sizeable part of a radiation department’s practice.

Before implementing on a routine large-scale basis, the impact of breast IMRT on cost and logistic issues needs to be ascertained. In order to suit the Canadian environment, we developed a template-based inverse optimization breast IMRT technique (TB-IMRT) to achieve the advantages of breast IMRT without being resource intensive. The purpose of this study is to compare efficiency of resources between CBR and breast TB-IMRT and to compare dose distributions between CBR and TB-IMRT to validate our technique.

## Methods

### Patients

Twenty patients (6 with right-sided and 14 with left-sided tumors) who had been previously treated with adjuvant external beam radiation therapy to the whole breast at the Fraser Valley Cancer Centre were randomly selected. They had either stage T1 or T2 breast cancer and had undergone breast conserving surgery. All patients had sentinel node biopsy and/or axillary nodal dissection. Chemotherapy, endocrine therapy and trastuzumab were given when indicated. Actual treatments for all 20 cases were carried out with CBR plans. Plans were then carried out retrospectively using the TB-IMRT technique for this same group of patients. Dose-fractionation varied from 42.5 Gy in 16 fractions (2.66 Gy per fraction) to 50 Gy in 25 fractions (2 Gy per fraction), given once per day, 5 days a week. Study approval was obtained from the University of British Columbia/B.C. Cancer Agency Institutional Review Board.

### Structure contouring for TB-IMRT and CBR

All patients had a simulation-CT scan performed in treatment position. All patients were supine with both arms extended above their head in a Vac-Lok™ cushion. A Civco MT-350 carbon fiber breast board with angles ranging from 5 to 20° from the couch was used. The surgical scar and clinical breast tissue was demarcated with a radio-opaque wire. Imaging was performed with a Philips Scanner (Brilliance CT, Big Bore, Philips, Cleveland, OH), and acquired in 2 mm slices from 6 cm superior to the clavicle to 8 cm inferior to breast tissue. CT images were exported to the treatment planning system, Eclipse version 10.0.28 (Varian Medical Systems, Palo Alto, CA). When the patient’s planning CT is imported into the Eclipse software, a body contour is generated, edited and post processed according to local procedure.

**Figure 1:**
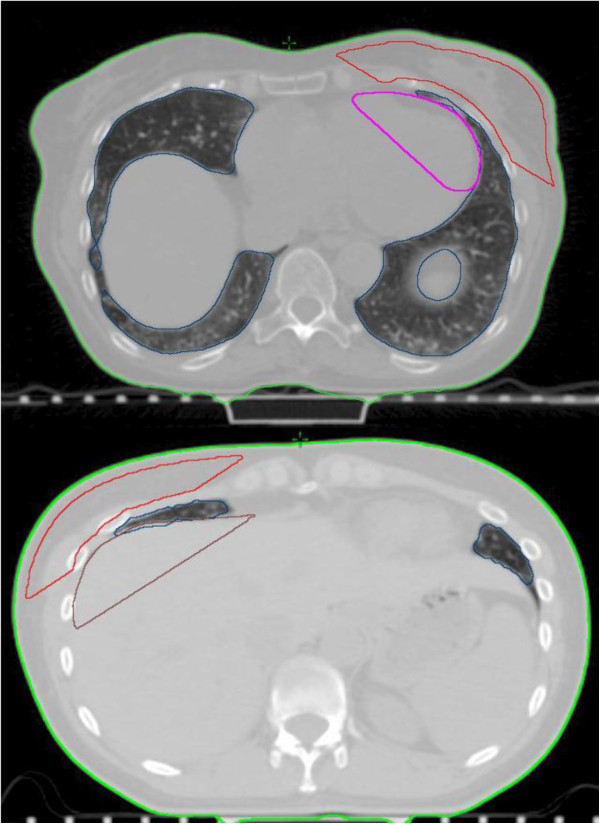
Portions of underlying critical organs (lung and liver for right-sided tumors and lung and heart for left-sided tumors) overlapping the posterior border of the tangents were contoured as structures to be excluded from the TVeval.

At our institution, the radiation oncologist adjusts beam placement based on the clinical borders of the breast volume using the radio-opaque markers placed in CT as well as the location of the lumpectomy cavity on simulation-CT. This was done for CBR and TB-IMRT. Typically the medial treatment beam entry point is at midline, the lateral beam is 2 cm posterior to palpable breast tissue, the superior limit of the beam is at or up to 1 cm below the suprasternal notch, and the inferior limit of the beam is 2 cm below palpable breast. The beam angles were thus selected by the treating radiation oncologist and were patient-dependent. The breast treated volume (TV) used for plan evaluation (TVeval) was defined with the aid of these tangential fields. The breast treated volume was thus defined as the volume encompassed by the tangential fields, placed by the radiation oncologist. In order to create a breast TVeval, portions of underlying critical organs (lung and liver for right-sided tumors and lung and heart for left-sided tumors) overlapping the posterior border of the tangents were contoured as structures to be excluded from the TVeval (Figure 1). The breast TVeval was delineated as 1.5 cm inside the superior and inferior field borders and 5 mm from the skin surface (using the body contour), lung, liver and/or heart. Lung, liver and heart contours were usually done to define the TV only and not used for dose reporting. The lung contours were created with an automatic tool in the Eclipse software and the heart or liver were contoured only in regions overlapping the tangent fields. A 31 mm circle is drawn on a central plane, at breast mid-separation, in air, at the superior aspect of the breast. This structure (skin flash) is added to extend the fluence boundary to a larger distance in order to make enough space for skin flash in the future, since the fluence matrix in Eclipse software has a spatial boundary near any structure with a lower constraint. For comparison purposes, the same delimitation of structures was used both for CBR and TB-IMRT.

### Planning technique

CBR was planned using wedged half beam blocked medial and lateral tangents with 6MV only or mixed with 18MV. The collimator angle is set off 90° to allow for use of enhanced dynamic wedging. When needed, subfields were used to minimize hot spots. Our TB-IMRT technique employed four half beam block fields with a collimator angle off 0°, to allow for the multileaf collimator to provide the optimal modulation. (The standard tangent beam arrangement used for CBR was used for TB-IMRT to minimize procedural change). Independent of dose prescription, two of the fields were modulated with 6 MV, while the other two fields remained open and utilized 6 or 18 MV. Fluence was generated with a dynamic multileaf collimator using Varian Millenium (sliding window technique) with 40 leafs of 0.5 cm width in the centre of the field and 40 leafs of 1 cm width in the outer region of the field.

### Inverse optimization for TB-IMRT

Two optimization templates were created with a script incorporated into the planning system (one for all fields with 6 MV and another for an unmodulated field with 18 MV energies). This defines the dose constraints for inverse planning. Values in the constraints within these 2 templates were adjusted if a different dose fractionation was used using a ratio of the 2 prescriptions. Calculations were done using the Eclipse analytical anisotropic algorithm version 10.0. Inverse optimization was used and each optimization used 30 iterations. Due to beam separation, we optimized the process and found that if the 6 MV template was used, one optimization was required, while the 18 MV template required two optimizations. The 18 MV template was used only when dose criteria were not met with the 6 MV template due to having three 18 MV machines only. Objectives were added to TVedit and to the skin flash structure only. The skin flash structure had 0 priority, allowing the fluence matrix to be extended without adding additional fluence there. No constraints were added to organs at risk.

### Treatment planning objectives and delivery

For each patient, the clinically used CBR plan was used. For TB-IMRT plans, objectives were based on the following criteria: at least 95% of the TVeval must be covered by 95% of the prescription, maximum dose must be less than 107% of the prescribed dose (107% is acceptable but must be less than 1.8 cc) while a point dose up to 110% is allowed, and mean TVeval dose should be 100%. Minimum dose should be 90%, but may be lower superficially. Treatment delivery was done on a Cl 21iX, Trilogy Linear Accelerator (Varian Medical Systems, Palo Alto, CA). The fluence patterns were manually extended from approximately the inside edge of the TV to beyond the skin surface using a 31 mm circle, to account for respiratory motion and variability in day-to-day setup.

Compared to CBR, TB-IMRT was a complete planning process change for our department. Our purpose was to ensure that each planning step be as standard as possible. Independent of the level of expertise of the planner, as long as he follows the detailed planning procedure, the plan should meet the planning goals. The TB-IMRT template thus includes three components: contouring, beam arrangements and optimization.

### Plan comparison parameters and statistics

For the purpose of this study, homogeneity and conformity, as well as mean and maximum dose were calculated for TVeval. The TV homogeneity index (HI) was defined as: HI = (D2% - D98%)/D_median _[10]. where D_median_ is the median dose to the TV, D2% and D98% are the maximum and minimum dose that covers 2% and 98% volume of the PTV on dose volume histogram [10]. The conformity index (CI) was calculated as CI = (VT_pres_/VT) x (VT_pres_/V_pres_) = V2T_pres_/(VT x V_pres_ ) where VT_pres_ is the target volume covered by the 95% isodose line, V_pres_ is the treated volume covered by the 95% isodose line and VT is the volume of target [11].

While they are not routinely contoured for TB-IMRT at our center, for dose-distribution analysis, ipsilateral and contralateral lungs, heart and contralateral breast were additionally contoured. Heart was contoured from its apex to the origin of the pulmonary arteries, excluding the pericardial fat and descending artery. The same anatomical landmarks used for ipsilateral breast TV determination were used for the contralateral breast. Normal tissues treated were compared on the basis of volume of ipsilateral lung receiving 5 Gy and 20 Gy or greater (V5 and V20, respectively); contralateral lung receiving 0.5 Gy and 5 Gy or greater (V0.5 and V5, respectively); volume of heart receiving 1 Gy, 5 Gy and 25 Gy (V1, V5 and V25, respectively); and volume of contralateral breast receiving 1 and 5 Gy (V1 and V5, respectively). A TV was delineated retrospectively on CBR plans, as well as aforementioned normal tissue structures for plan comparisons. Timing of each CBR plan for workload impact evaluation did not include time to contour these structures. Timing of each TB-IMRT plan included time to contour structures needed for TV determination and optimization. Comparisons between CBR and TB-IMRT were made using the paired *t* test (two-sided) for each parameter. Statistical comparisons were performed using Statistica (version 7.1) (StatSoft, Tulsa, OK). Results were considered significant for p-values below 0.05.

### Workload impact

The following parameters were used to determine the impact of both the CBR and TB-IMRT treatment techniques on resources: time to plan (time for dosimetry planning for CBR and for TB-IMRT time for contouring of structures and plan optimization), time to deliver treatment and time to perform quality assurance. Time to plan for CBR was evaluated by the dosimetrist assigned to the plan, while TB-IMRT plans were timed and generated by the same individual (SKAN). Treatment delivery time was evaluated in the same 20 patients with CBR plans who had actual treatments carried out. In addition, treatment delivery time was measured in another set of patients who had breast plans carried out with a TB-IMRT technique. Treatment delivery time did not include port films or other image guidance.

For both CBR and TB-IMRT plans, IMSURE QA™ Software was used for patient-specific quality assurance (QA) for the clinic cases. Patient-specific QA was performed using an in-house program, Epidose, for the 20 TB-IMRT plans [12]. Epidose uses portal images to reconstruct a 3D dose distribution in a phantom and compares this to the dose distribution generated by the treatment planning system.

## Results

### Workload impact

For the 20 patients planned, best CBR plans took an average 39.0 min to be produced (15–70). For TB-IMRT plans, structure contouring to determine TV took an average 6.5 min (2.9-10.2). Planning time then took an average 7.7 min (4.8-11.5). In total, TB-IMRT plans were done in 14.0 min (7.9-19.4), which was less than CBR planning time (14.0 vs 39.0 min, p < 0.001). 15 patients with CBR needed 18 MV and 11 of these were planned successfully with IMRT using only 6 MV. The average total beam-on time for these 20 patients with CBR plans were similar to another set of 20 patients with TB-IMRT plans (6.0 vs 7.8 min, p = 0.13).

For the 20 patients in which Epidose QA was performed all 20 of the TB-IMRT plans had 90% agreement of points comparisons between the reconstructed 3D dose distribution in a phantom using 3% dose and 3 mm position tolerances. The generated plan thus passed the patient QA dose test.

### Target volume coverage

Comparison of planning target volume and dose to organs at risk parameters between TB-IMRT and CBR for the 20 patients is shown in Table 1. TB-IMRT provided better homogeneity index (0.096 vs 0.124, p < 0.001, with ideal value 0) and conformity index (0.68 vs 0.59, p = 0.003, with ideal value 1). Maximal and mean dose were similar between TB-IMRT and CBR plans.

**Table 1:** Plan comparison parameters* between TB-IMRT and CBR techniques

**Structure**	**Parameters**	**TB-IMRT**	**CBR**	***p *value **^**†**^
TV	CI	0.68 (0.083)	0.59 (0.15)	0.003
	HI	0.096 (1.2)	0.13 (2.2)	< 0.001
	Mean dose (%)	100.4 (0.80)	99.8 (2.6)	0.37
	Maximal dose (%)	106.7 (0.85)	106.3 (0.97)	0.12
Ipsilateral lung	V5 (%)	22.2 (8.0)	20.3 (8.2)	0.09
	V20 (%)	12.5 (5.0)	12.9 (5.2)	0.41
Contralateral lung	V0.5 (%)	4.7 (8.1)	10.3 (9.5)	0.006
	V5 (%)	Negligible	Negligible	NS
Heart	V1 (%)	33.5 (2.2)	39.1 (21)	0.006
	V5 (%)	4.91 (6.5)	4.7 (5.1)	0.67
	V25 (%)	2.2 (3.8)	2.2 (2.5)	0.32
Contralateral breast	V1 (%)	1.4 (2.1)	6.2 (4.4)	0.0003
	V5 (%)	Negligeable	Negligeable	NS

*Abbreviations:* D2% and D98% are the maximum and minimum dose that covers 2% and 98% volume of the PTV on dose volume histogram; *TB-IMRT* = template-based intensity-modulated radiation therapy; *NS* = non significant; *TV* = treated volume; *Vn (5)* = percent volume receiving *n* Gy or greater.

*Average values (standard deviation) for the 20 patients.

^†^ Two-sided paired *t* test.

### Normal tissue irradiation

Volume of ipsilateral lung receiving 20 Gy (V20- ipsilateral lung), and volume of heart receiving 25 Gy (V25- heart) were similar between CBR and IMRT plans. There is a trend for higher V5-ipsilateral lung with IMRT (22.2% vs 20.3%, *p* = 0.08) and IMRT yielded plans with lower V0.5-contralateral lung (4.67% vs 10.3%, *p* = 0.006), lower V1-heart (33.5% vs 39.1%, *p* = 0.006) and lower V1-contralateral breast (1.4% vs 6.2%, p = 0.0003).

## Discussion

Breast TB-IMRT resulted in faster planning and similar treatment time compared to CBR. TB-IMRT also permitted less use of high energy beams, useful in departments with predominance of monoenergy linacs. Furthermore, TB-IMRT plans improved homogeneity and conformity indices and yielded similar or better dose-volume histograms for lung and heart receiving a high dose.

In order to validate our technique, dose-volume histograms were generated between CBR and TB-IMRT plans for comparison. All of our TB-IMRT plans showed improved coverage and homogeneity with similar or better parameters for organs at risk, consistent with previously published data [4,5,13]. Randomized trials of IMRT versus non-IMRT techniques for breast cancer have reported that improved plans can, in turn, correlate with a decrease in acute and chronic skin toxicity, including moist desquamation, fibrosis and late changes on cosmesis [6,7,14]. Some plans in those studies were done using a forward-planned IMRT technique. Some have suggested better target homogeneity with inverse-planned over forward-planned breast IMRT [15]. We aimed to put into a template a less labour-intensive technique which is less dependent on the planner’s experience. Thus, inverse optimization with minimal objectives and without constraints was favoured. In our study, planning time for TB-IMRT was significantly shorter compared to CBR. While varying IMRT techniques have been reported, some have also used two open fields with segments of IMRT within the same tangential fields to function as compensators to reduce treatment planning time [3,5,13,16-19].

Planning and delivery times are usually reported as a median or average value or as an approximation, with few quoting a range of time. Farace et al. tested hybrid IMRT using semi-automated methods and use of an optimization volume as the target objective [18]. Goals were achieved in 61 patients with 2 conventional open and 2 IMRT tangents, similar to our technique. Total planning time was on average 10 min. Mayo et al. compared dose-volume histograms for five techniques for 10 breast patients: CBR, forward-planned field-in-field IMRT, IMRT only tangents, conventional open plus IMRT tangents similar to our technique, and finally, conventional open plus IMRT tangents with 2 anterior oblique IMRT beams [13]. The conventional open plus IMRT tangents (what they term “hybrid-IMRT”) achieved the best dose distribution, and was similar to field-in-field IMRT yet required only approximately 15 min to optimize. These authors used normal tissue contouring (e.g., lung and heart) and delineation of a breast CTV, which required additional effort from both the physician and dosimetrist. However, they stated that it took less than 10 min to define breast CTV after some experience, although this learning curve time was not specified in the report. In Fong et al.’s planning study, comparisons between several IMRT techniques and CBR were performed. Without mentioning specific times, they stated that tangential beam IMRT was less difficult and less time consuming than multi-field IMRT [17]. Descovich et al. investigated the planning efficiency of direct aperture optimization IMRT (where the delivery parameters, such as number of segments, shapes and weights are directly considered during optimization) versus forward planning IMRT using two tangential beams for whole breast [16]. Forward planning took 60–90 min, increasing to 3–5 h for a less experienced planner. Direct aperture optimization IMRT took 20–30 min to generate.

A source of weakness in this study which could have affected measurements, was that time to plan for CBR was measured by the dosimetrist assigned to the plan, while TB-IMRT plans were timed and generated by the same individual (SKAN). Nevertheless, while our dosimetrists are experienced in CBR planning, TB-IMRT was a new planning technique in our department. Interestingly, there was an almost non-existent learning curve for TB-IMRT plans contoured and optimized by the same individual. In addition, since it was not feasible to compare treatment times between CBR and TB-IMRT in the same set of patients, we used another set of 20 patients who were planned using TB-IMRT for treatment time comparisons. This might also have affected measurements, although both set of patients had comparable characteristics.

Our planners now use templates for breast TB-IMRT to replace CBR, with predetermined parameters for optimization, to obtain desired fluence patterns and beam weighting for each individual patient. The use of templates to facilitate IMRT planning for increased efficiency has been published for other disease sites [20,21]. To our knowledge, we are the first to document the use of templates for inverse planning breast IMRT. Contouring only what was necessary for planning can be done easily, and when parameters are set in advance into a template, optimization can become less labour intensive. Our TB-IMRT technique avoided unnecessary time spent contouring structures for inverse optimization. We found small, albeit statistically significant improvements in some parameters, such as lower dose to contralateral lung, heart and contralateral breast, with a trend to lower dose to ipsilateral lung. This is likely to be of little clinical significance, and contouring structures for dose reporting is not standard for TB-IMRT of the breast in our department.

Applying a template for inverse optimization permits IMRT use, yet is less dependent on the planner’s experience with dose optimization. The learning trajectory for implementing TB-IMRT is very short, and the same software tools with no additional hardware can also be used. TB-IMRT allowed a significant (11 out of 15) number of plans to be done using 6 MV only, permitting transfer to underutilized single energy accelerators. Also, neutron background is eliminated if only 6 MV photons can be used alone without higher energy photons. Equal treatment times between TB-IMRT and CBR do not affect treatment workload and patient convenience. Finally, since there is no increase in time for plan QA, physicist workload would not suffer. Taken together, these findings support implementation of TB-IMRT in our department, and can easily be transferable to other departments as well.

## Conclusions

TB-IMRT provided reduction of planning time and use of high energy beams, with similar treatment times and equal or better plans compared to CBR. This technique permits rational use of resources with a low learning curve, and can be done with existing equipment and personnel.

## Consent

Written informed consent was obtained from the patient for publication of this report and any accompanying images.

## Competing interests

The authors declare that they have no competing interests.

## Authors’ contributions

SKAN drafted the manuscript; participated in the design of the study, carried out the contouring and planning of all TB-IMRT cases and performed the statistical analysis. FC participated in the design of the study and co-designed the TB-IMRT template. RR co-designed the TB-IMRT template. SK, KK, CE, PS, JH, CC, BS, WK participated in the design and coordination of TB-IMRT. SK, CE, KK, FC, SK, WK helped draft the manuscript. All authors read and approved the final manuscript.
